# Supplementary material for: HIVprotI: an integrated web based platform for prediction and design of HIV proteins inhibitors
Source: J Cheminform. 2018 Mar 9;10:12. doi: 10.1186/s13321-018-0266-y (PMC5845081; doi:10.1186/s13321-018-0266-y)
Supplement: Supplementary file 2 — Additional file 2. Source code of HIVProtI web server. [file 13321_2018_266_MOESM2_ESM.zip › HIVprotI_Source-code/hivprotI/weka-3-6-6/doc/weka/core/EditDistance.html]

EditDistance


---


|  |  |  |
| --- | --- | --- |
| **Overview** \| **Package** \| **Class** \| **Tree** \| **Deprecated** \| **Index** \| **Help** \| **Weka's home** | |  |
| **PREV CLASS**   **NEXT CLASS** | **FRAMES**    **NO FRAMES**     **All Classes** |  |
| SUMMARY: NESTED \| FIELD \| CONSTR \| METHOD | DETAIL: FIELD \| CONSTR \| METHOD |  |


---


## weka.core Class EditDistance

```
java.lang.Object
  weka.core.NormalizableDistance
      weka.core.AbstractStringDistanceFunction
          weka.core.EditDistance
```

**All Implemented Interfaces:**: java.io.Serializable, DistanceFunction, OptionHandler, RevisionHandler

---

``` public class EditDistance extends AbstractStringDistanceFunction ```

Computes the Levenshtein edit distance between two strings.

**Version:**
:   $Revision: 1.2 $

**Author:**
:   Bruno Woltzenlogel Paleo

**See Also:**: Serialized Form

---

**Field Summary**

**Fields inherited from class weka.core.NormalizableDistance**

`R_MAX, R_MIN, R_WIDTH`


**Constructor Summary**

`EditDistance()`

`EditDistance(Instances data)`


| **Method Summary** | |
| --- | --- |
| `java.lang.String` | `getRevision()`             Returns the revision string. |
| `java.lang.String` | `globalInfo()`             Returns a string describing this object. |

**Methods inherited from class weka.core.AbstractStringDistanceFunction**

`distance`

**Methods inherited from class weka.core.NormalizableDistance**

`attributeIndicesTipText, distance, distance, distance, dontNormalizeTipText, getAttributeIndices, getDontNormalize, getInstances, getInvertSelection, getOptions, getRanges, initializeRanges, initializeRanges, initializeRanges, initializeRangesEmpty, inRanges, invertSelectionTipText, listOptions, postProcessDistances, rangesSet, setAttributeIndices, setDontNormalize, setInstances, setInvertSelection, setOptions, toString, update, updateRanges, updateRanges, updateRanges, updateRangesFirst`

**Methods inherited from class java.lang.Object**

`equals, getClass, hashCode, notify, notifyAll, wait, wait, wait`

**Constructor Detail**

### EditDistance

```
public EditDistance()
```

---


### EditDistance

```
public EditDistance(Instances data)
```


**Method Detail**

### globalInfo

```
public java.lang.String globalInfo()
```

:   Returns a string describing this object.

    :   **Specified by:**: `globalInfo` in class `NormalizableDistance`
    :   **Returns:**: a description of the evaluator suitable for displaying in the explorer/experimenter gui

---


### getRevision

```
public java.lang.String getRevision()
```

:   Returns the revision string.

    :   **Returns:**: the revision


---


|  |  |  |
| --- | --- | --- |
| **Overview** \| **Package** \| **Class** \| **Tree** \| **Deprecated** \| **Index** \| **Help** \| **Weka's home** | |  |
| **PREV CLASS**   **NEXT CLASS** | **FRAMES**    **NO FRAMES**     **All Classes** |  |
| SUMMARY: NESTED \| FIELD \| CONSTR \| METHOD | DETAIL: FIELD \| CONSTR \| METHOD |  |


---
